# Improved Production of Majority Cellulases in *Trichoderma reesei* by Integration of *cbh1* Gene From *Chaetomium thermophilum*

**DOI:** 10.3389/fmicb.2020.01633

**Published:** 2020-07-14

**Authors:** Xianzhang Jiang, Jiawen Du, Ruonan He, Zhengying Zhang, Feng Qi, Jianzhong Huang, Lina Qin

**Affiliations:** ^1^National Joint Engineering Research Center of Industrial Microbiology and Fermentation Technology, College of Life Sciences, Fujian Normal University, Fuzhou, China; ^2^Provincial University Key Laboratory of Cellular Stress Response and Metabolic Regulation, College of Life Sciences, Fujian Normal University, Fuzhou, China

**Keywords:** *Trichoderma reesei*, thermophilic fungi, *Chaetomium thermophilum*, cellobiohydrolase I, lignocellulase, biomass, cellulase

## Abstract

Lignocellulose is an abundant waste resource and has been considered as a promising material for production of biofuels or other valuable bio-products. Currently, one of the major bottlenecks in the economic utilization of lignocellulosic materials is the cost-efficiency of converting lignocellulose into soluble sugars for fermentation. One way to address this problem is to seek superior lignocellulose degradation enzymes or further improve current production yields of lignocellulases. In the present study, the lignocellulose degradation capacity of a thermophilic fungus *Chaetomium thermophilum* was firstly evaluated and compared to that of the biotechnological workhorse *Trichoderma reesei.* The data demonstrated that compared to *T. reesei, C. thermophilum* displayed substantially higher cellulose-utilizing efficiency with relatively lower production of cellulases, indicating that better cellulases might exist in *C. thermophilum*. Comparison of the protein secretome between *C. thermophilum* and *T. reesei* showed that the secreted protein categories were quite different in these two species. In addition, to prove that cellulases in *C. thermophilum* had better enzymatic properties, the major cellulase cellobiohydrolase I (CBH1) from *C. thermophilum* and *T. reesei* were firstly characterized, respectively. The data showed that the specific activity of *C. thermophilum* CBH1 was about 4.5-fold higher than *T. reesei* CBH1 in a wide range of temperatures and pH. To explore whether increasing CBH1 activity in *T. reesei* could contribute to improving the overall cellulose-utilizing efficiency of *T. reesei*, *T. reesei cbh1* gene was replaced with *C. thermophilum cbh1* gene by integration of *C. thermophilum cbh1* gene into *T. reesei cbh1* gene locus. The data surprisingly showed that this gene replacement not only increased the cellobiohydrolase activities by around 4.1-fold, but also resulted in stronger induction of other cellulases genes, which caused the filter paper activities, Azo-CMC activities and β-glucosidase activities increased by about 2.2, 1.9, and 2.3-fold, respectively. The study here not only provided new resources of superior cellulases genes and new strategy to improve the cellulase production in *T. reesei*, but also contribute to opening the path for fundamental research on *C. thermophilum.*

## Introduction

Plant biomass from agriculture and forestry is one of the most abundant resources on the earth and it has been considered as a promising material for producing renewable biofuels and other value-added bio-products (Himmel et al., 2007). One of the bottlenecks of this process is the enzymatic degradation of biomass-derived polysaccharides. Currently, the filamentous fungi *Trichoderma*, *Aspergillus*, and *Penicillium* species are the most commonly used producer for lignocellulases in industry (Kubicek et al., 2014). However, the optimum temperature of enzymes from these species is normally from 30 to 50°C at which the efficiency of biomass polysaccharides saccharification is very low. Many thermophilic fungi can secrete thermostable biomass-degrading enzymes including lignocellulases, proteases, amylases, laccases, chitinases, lipases, and esterases, which holds a great promise in industrial applications (DeCastro et al., 2016). However, due to the lack of genetic tools for strain engineering in thermophilic species, progress toward the improvement of their enzyme production has been hampered. One way to overcome these drawbacks is the introduction of lignocellulases encoding genes from thermophilic fungi into mesophilic species, in which a variety of genetic tools have been developed, to combine the advantages of mesophilic and thermophilic properties.

The soft-rot fungus *Trichoderma reesei* is a model organism for plant biomass degradation and widely used in industry for the production of cellulases and xylanases due to the large capacity of hydrolytic enzymes secretion (Bischof et al., 2016). After rounds of random mutagenesis, several industrial strains can secrete lignocellulases over 100 g/L culture (Cherry and Fidantsef, 2003). Besides its large protein secretion capacity, *T. reesei* naturally has the ability to form disulfide bonds and to glycosylate proteins (Singh et al., 2015). In addition, *T. reesei* can grow on inexpensive substrate such as lignocellulosic waste materials and many secreted enzymes from *T. reesei* are under the GRAS status (Generally Recognized as Safe) (Sewalt et al., 2016). These properties have promoted efforts to develop *T. reesei* as an excellent host for production of lignocellulases and recombinant proteins. Many studies have shown that the lignocellulose degradation ability could be improved by recombinant expression of biomass degrading enzymes from different organisms in *T. reesei* (Zhang et al., 2010; Dashtban and Qin, 2012; Treebupachatsakul et al., 2015).

*Chaetomium thermophilum*, an ascomycete thermophilic species, commonly exists in the heating phase of composts with an optimum growth temperature around 50°C (Kellner et al., 2016). The release of *C. thermophilum* genome database (Bock et al., 2014) and several biotechnological studies indicate that there are valuable biomass degradation associated enzymes existed in *C. thermophilum* (Voutilainen et al., 2008; Li et al., 2010). However, the secreted enzyme components and their properties in *C. thermophilum* have been rarely studied. In the present study, the capability of lignocellulose degradation of *C. thermophilum* was evaluated and compared to that of *T. reesei.* Besides, the enzyme properties of the major cellulase cellobiohydrolase I (CBH1) from *T. reesei* and *C. thermophilum* were compared. Moreover, to explore whether increasing CBH1 activities in *T. reesei* cellulases complex could contribute to improving the cellulose degradation efficiency, the native *T. reesei cbh1* gene was replaced with *C. thermophilum cbh1* gene in *T. reesei*. The CBH activities, filter paper activities, endoglucanases activities, and β-glucosidase activities, along with the transcriptional levels of several cellulases genes including *eg1*, *eg2*, *cbh2*, and *bgl1*, as well as the transcriptional factors (TFs) *xyr1*, *cre1*, and *ace3* of the resultant strains have been investigated. This study may provide a new resource of superior cellulases genes and a new strategy to improve the efficiency of biomass saccharification by *T. reesei*.

## Materials and Methods

### Microbial Strains and Growth Conditions

*Escherichia coli* Trans1-T1 used for recombinant plasmid construction was bought from TransGen Biotech. *T. reesei* Tu6 strain (ATCC MYA-256) was used as control to evaluate lignocellulose degradation efficiency of *C. thermophilum*. *T. reesei* Tu6Δ*ku70* strain used as host strain to construct indicated recombinant strains was kindly provided by Dr. Monika Schmoll (AIT Austrian Institute of Technology, Austria). *T. reesei* strains Tr-cT*cbh1*, Tr-cC*cbh1*, and Tr-Ct*cbh1* were constructed in this study and described below. *C. thermophilum* (CGMCC 3.17990) was provided by China general microbiological culture collection center. *T. reesei* strains were grown for 5 to 6 days at 28°C on potato dextrose agar plates (PDA) or PDA supplemented with 5 mM uridine when necessary. Minimal medium (Penttilä et al., 1987) was applied as a selective medium for the screening of *T. reesei* transformants. *C. thermophilum* strain was grown for 5–7 days at 50°C on PDA slants.

For protein secretion and enzyme activities analysis, *T. reesei* conidia were firstly inoculated into 100 mL liquid minimal medium with 2% glucose as the carbon source (MM+2% glucose) at 10^6^ conidia/mL and grown at 28°C on a rotary shaker for 48 h at 200 rpm, the mycelia were then filtered through 200 mesh sifter (30 μm pore diameter) and washed twice with carbon-free medium. About 2.2 g of this wet mycelia was added into 50 mL of fresh cellulase-inducing minimal medium in which 2% glucose (w/v) was substituted with 2% Avicel or 2% sugarcane bagasse and grown at 200 rpm at 28°C in consistent dark. For analysis of *C. thermophilum*, 7 days of *C. thermophilum* culture grown on a slant (18 mm × 180 mm) was scraped and first inoculated into *Neurospora* Vogel’s minimal medium (VMM) (Vogel, 1956) with 2% glucose as carbon source and grown at 50°C on a rotary shaker (200 rpm) for 36 h. The cultures were then filtered and about 2.2 g of the wet mycelia was respectively transferred into 50 mL VMM media with 2% Avicel or 2% sugarcane bagasse as carbon source and grown at 50°C on a rotary shaker (200 rpm) for indicated time in the natural light.

### Transformation of *T. reesei* Strains by Electroporation

The electroporation transformation method was based on the protocol described in Schuster et al. (2012) with some optimization. In brief, fresh *T. reesei* conidia (within 7 days) grown on a 90 mm agar plate was harvested and washed three times using 1.1 M ice cold sorbitol to make conidia suspension at the concentration of 10^8^ conidia/mL. A total of 1–2 μg of transformed DNA in a final volume of 10 and 90 μL of sorbitol washed conidia suspension was put into pre-chilled electroporation cassette. Gene Pulser Xcell electroporation system (Bio-Rad, United States) was used for electroporation with the set of 1.6 kV, 600 Ω, 25 μF. After electroporation, 900 μL of 1.1 M icy cold sorbitol was immediately added into the electroporation reaction mix before transferring into 9 mL of YPD (1% yeast extract, 2% peptone, 1% dextrose). The whole mixture was incubated for 12 h at 30°C and centrifuged at 2000 × g for 5 min. The electroporated conidia were re-suspended in 500 μL of YPD and added with 14 mL of top MM agar media. The whole mixture was spread over 2–3 bottom MM agar plates and incubated at 30°C for 5–7 days.

### Construction of *T. reesei* Recombinant Strains

C*pyr4* series strains were constructed by transforming strain Tu6Δ*ku70* with plasmid pSK-*pyr4* (Qin et al., 2012).

Tr-cT*cbh1* was constructed by transforming strain Tu6Δ*ku70* with a DNA fragment including *pyr4* gene expression cassette and cDNA1 promoter (936 bp upstream of the start codon of cDNA1 gene) flanked with 2 kb upstream of *cbh1* promoter (1.5 kb upstream of the start codon of *cbh1* gene) and 2 kb downstream of *cbh1* promoter. The plasmid pTrcT*cbh1* used for generating Tr-cT*cbh1* transformants was constructed by ligation of the above fragments into a backbone from plasmid pBluescript SK (+). The primer pair pTrcT*cbh1-*F1F/R was used for amplifying 2 kb fragment of the upstream of *cbh1* promoter. The primer pair pTrcT*cbh1-*F2F/R was used to amplify the *pyr4* gene expression cassette. The primer pair pTrcT*cbh1-*F3F/R was used for amplifying cDNA1 promoter. The primer pair pTrcT*cbh1-*F4F/R was used for amplifying 2 kb downstream of *cbh1* promoter. The primer pair pTrcT*cbh1-*F5F/R was used for amplifying the vector backbone from plasmid pBluescript SK (+).

Tr-cC*cbh1* strain was generated by transforming strain Tu6Δ*ku70* with a fragment containing the expression cassette of *pyr4* gene, cDNA1 promoter and cDNA sequence of *C. thermophilum cbh1* gene (Accession No: AM711862.1), flanked with 2 kb upstream of the *cbh1* promoter and 2 kb downstream of *T. reesei cbh1* gene (Accession No: XM_006969162). The plasmid pTrcC*cbh1* used for generating Tr-cC*cbh1* transformants was constructed by ligation of the fragments of cDNA sequence of *C. thermophilum cbh1* gene and 2 kb downstream of *T. reesei cbh1* gene into the vector backbone from the plasmid pTrcT*cbh1.* The primer pair pTrcC*cbh1-*F1F/R was used for amplifying cDNA sequence of *C. thermophilum cbh1* gene. The primer pair pTrcC*cbh1-*F2F/R was used for amplifying the fragment of 2 kb downstream of *T. reesei cbh1* gene. The primer pair pTrcC*cbh1-*F3F/R was used for amplifying the vector backbone which included 2 kb fragment of the upstream of *cbh1* promoter, *pyr4* gene expression cassette, and cDNA1 promoter.

Tr-Ct*cbh1* was created by co-transforming strain Tu6Δ*ku70* with linearized plasmid pSK-*pyr4* (Qin et al., 2018) and a fragment including cDNA sequence of *C. thermophilum cbh1* gene, flanked with 2 kb upstream and downstream of *T. reesei cbh1* gene. The plasmid pTrCt*cbh1* used for generating Tr-Ct*cbh1* transformants was constructed by ligation of 2 kb upstream of *T. reesei cbh1* gene and cDNA sequence of *C. thermophilum cbh1* gene into the vector backbone from plasmid pTrcC*cbh1.* The primer pair pTrCt*cbh1-*F1F/R was used for amplifying 2 kb upstream of *T. reesei cbh1* gene. The primer pair pTrCt*cbh1-*F2F/R was used for amplifying cDNA sequence of *C. thermophilum cbh1* gene. The primer pair pTrCt*cbh1-*F3F/R was used for amplifying the vector backbone from plasmid pTrcC*cbh1* which included 2 kb downstream of *T. reesei cbh1* gene.

All types of transformants were selected on minimal media without adding uridine and tested for genotypes by diagnostic PCR. The sequence of all the primers used in this study was listed in the Supplementary Table S1.

### Determination of Biomass in Culture Including Insoluble Cellulose

As described before (Stappler et al., 2017), the abundance of biomass accumulation in the culture containing insoluble cellulose was determined by the measurement of the intracellular protein concentration of mycelia. The mycelia in culture were filtered using a vacuum filter system and added 1 mL 0.1 M NaOH and lysed using a mini-bead beater (Biospec Products, Bartlesville, OK, United States) with 0.5 mm diameter glass beads. The mixture was incubated for 1 h at room temperature and centrifuged for 5 min at 12,000 rpm and the supernatants were transferred to new tubes. The protein concentration in the supernatants was measured by using the Bradford Protein Assay.

### Protein Concentration and Enzyme Activities

Protein concentration in supernatants of all cultures was determined by Bradford method by following the instruction (Bio-Rad Protein Assay). Cellobiohydrolase (CBH) activities of Tr-cT*cbh1*, Tr-cC*cbh1*, and C*pyr4* series strains under glucose were measured using 1 mM soluble 4-methylumbelliferyl-b-D-cellobiose (MUC, Sigma) as a substrate following methods described in Wu et al. (2017). In brief, 10 μL of culture supernatant and 40 μL of 1 mM MUC in 50 mM sodium acetate buffer (pH 5) were incubated together at 50°C for 15 min by using thermocycler. After 15 min, the temperature was raised to 95°C for 5 min to deactivate the enzyme. After cooling down to room temperature, the fluorescence was measured at 445 nm with excitation with 365 nm. CBH activities of Tr-Ct*cbh1* and C*pyr4* series strains under cellulose were measured using the same substrate, but the method was as described as in Bailey and Tähtiharju (2003). CBH1 activity was calculated by subtraction of β-glucosidase and endoglucanase activity. One unit of CBH activity was defined as the amount of enzyme necessary to release 1 μmol methyl umbelliferone in one minute. The total cellulase activities in the supernatants were measured by filter paper activity using Whatman No. 1 filter paper as the substrate (Ghose, 1987). Endoglucanase activity was determined by using azo-carboxymethyl cellulose (Azo-CM-Cellulose; Megazyme) as the substrate and following the manufacturer’s specifications. β-glucosidase activity was measured by using 4-nitrophenyl -D-glucopyranoside (pNPG) as the substrate and following the method described in Tian et al. (2009). In brief, 50 μL of 10 × diluted culture supernatant was added into 200 μL of 500 μM pNPG in 50 mM sodium acetate buffer, pH 5.0. The mixture was incubated at 50°C for 10 min and terminated by adding 750 μL of 1 M Na_2_CO_3_. After cooling down to room temperature, the absorbance at 400 nm was measured. One unit of β-glucosidase activity was defined as the amount of enzyme necessary to release 1 μmol p-nitrophenol in 1 min.

### Optimal pH and Temperature Analysis of the Recombinant CBH1

The optimum pH was determined by measuring the enzyme activities using Glycine-HCl (pH 2.0–3.0, 50 mM), Citric buffer (pH 3.0–6.0, 50 mM), phosphate buffer (pH 6.0–8.0, 200 mM) and Glycine-NaOH (pH 9.0–10.0, 50 mM) at 50°C. A total of 25 μL of filtered culture supernatant was added into 75 μL of the each of the above buffers with 1 mM MUC as substrate and incubated at 50°C for 1 h and then transferred to 95°C for 5 min to deactivate the enzyme. The enzyme activities were calculated by the absorbance under 445 nm emission and 365 nm excitation after incubation. The pH stability was assayed by incubating the enzyme at different pH range from pH 2.0–9.0 for 2 h at room temperature. Then the samples were diluted 10-fold in 50 mM citric buffer, pH 4.0 before measuring the residual activity. The optimum temperature of recombinant CBH1 was determined between 20 and 80°C in 50 mM citric buffer, pH 4.0. Thermal stability was investigated by incubating the enzyme at a different temperature ranging from 20 to 80°C for 30 min in 50 mM citric buffer, pH 4.0. The remaining activities were measured under standard conditions.

### Secretory Protein Analysis by Liquid Chromatography-Mass Spectrometry

The supernatants of 120 h of *C. thermophilum* and *T. reesei* culture under Avicel were used to run SDS-PAGE firstly. The SDS-PAGE gels were subsequently cut and digested by trypsin and then analyzed using an EASY-nLC 1000 liquid chromatograph which was connected in line with an Orbitrap Fusion Tribrid mass spectrometer equipped with a nanoelectrospray ionization (nanoESI) source (Thermo Fisher Scientific, Waltham, MA, United States). The peptides were loaded onto a trap column (C18, 5 μm particles, 100 μm ID, 3 cm length, Dr. Maisch GmbH) and separated using an analytical column (C18, 3 μm particles, 75 μm ID, 15 cm length, Dr. Maisch GmbH) at a flow rate of 400 nL/min with a 60 min LC gradient composed of Solvent A [0.1% formic acid (v/v)] and Solvent B [acetonitrile, 0.1% formic acid (v/v)]. The gradient was 3–8% B for 3 min, 8–23% B for 45 min, 23–35% B for 9 min, 35–80% B for 2 min, and finally 80% B for 1 min. Full-scan mass spectra were acquired in the positive-ion mode over the m/z range from 350 to 1550 using the Orbitrap mass analyzer in profile format with a mass resolution setting of 120,000. In the data-dependent mode, the top-speed mode was selected with the most intense ions exceeding an intensity threshold of 5000 counts from each full-scan mass spectrum for tandem mass spectrometry (MS/MS) analysis using HCD activation type with 33% collision energy. MS/MS spectra were acquired in centroid format using an ion trap detector. Real-time charge state screening was enabled to exclude unassigned and 1+ charge states from MS/MS analysis. The exclusion duration in dynamic exclusion was set as 20 s. Raw data were searched against the amino acid sequences of *T. reesei* or *C. thermophilum* using Mascot software (version 2.5).

### HPAEC-PAD Analysis of Released Glucose and Cellobiose From Filter Paper

A total of 500 μL of appropriately diluted hydrolysates from filter paper was analyzed on a Thermo Scientific Dionex ICS-5000 high-performance liquid chromatography (Dionex Corporation, Sunnyvale, CA, United States) instrument equipped with a CarboPac PA100 guard column (4 × 50 mm) and an analytical column (4 × 250 mm) with a flow rate as 1 mL/min at 22°C. The samples were resolved in a mobile phase of 100 mM NaOH. Glucose and cellobiose were used as standards.

### Quantitative RT-PCR Analysis

An aliquot of 2 × 10^6^ mL of *T. reesei* conidia were first inoculated into 100 mL MM+2% glucose media and grown at 28°C on a rotary shaker for 48 h at 200 rpm, the mycelia were then filtered through 200 mesh sifter (30 μm pore diameter) and washed twice with carbon-free MM media. A total of 2.2 g of the filtered wet mycelia were transferred to MM+1% Avicel medium and cultured at 28°C in the constant dark at 200 rpm for 48 h. Biological triplicates were prepared for each strain. Total RNA was isolated from samples taken at 12, 24, and 48 h of shift using zirconia/silica beads and a Mini-Beadbeater with 1 mL TRIzol reagent (from Invitrogen). RNA purification and DNase I treatment were performed using Easy Pure RNA Purification Kit (from TransGen Biotech). qRT-PCR was performed using TransScript Green One-Step qRT-PCR SuperMixKit (from TransGen Biotech) on the LightCycler 96 instrument (from Roche Life Science). Primer sequences used for amplification are described in Supplementary Table S1. The relative transcriptional levels of the tested genes were normalized to the expression level of the actin gene and calculated by 2^–Δ^*^*C*^*^*t*^ as a relative expression level.

### Statistical Significance Tests

For all the experiments, three biologically replicated strains and three technical replicates for each strain were set for statistical analysis. Statistical significance was determined by *t*-test analysis by the false discovery rate (FDR) approach by using the Prism GraphPad software.

## Results

### *Chaetomium thermophilum* Displayed Greater Lignocellulose Utilization Ability Compared to *T. reesei*

To search for thermophilic lignocellulases with outstanding enzyme properties, the cellulose degradation capacity of the thermophilic fungus *C. thermophilum* was evaluated and compared to that of *T. reesei*. To alleviate potential effects caused by differential spore germination efficiency, *C. thermophilum* CGMCC 3.17990 and *T. reesei* strain Tu6 were firstly grown in medium containing glucose as the sole carbon source and the same amount of wet mycelia (about 2.2 g) were then transferred into medium containing Avicel or sugarcane bagasse as the carbon source. The biomass accumulation, the extracellular protein concentration, and the filter paper activities in the supernatant were measured at different time points. The abundance of biomass accumulation in the culture containing insoluble carbon source Avicel or sugarcane was determined by the measurement of the intracellular protein concentration of mycelia.

**FIGURE 1:**
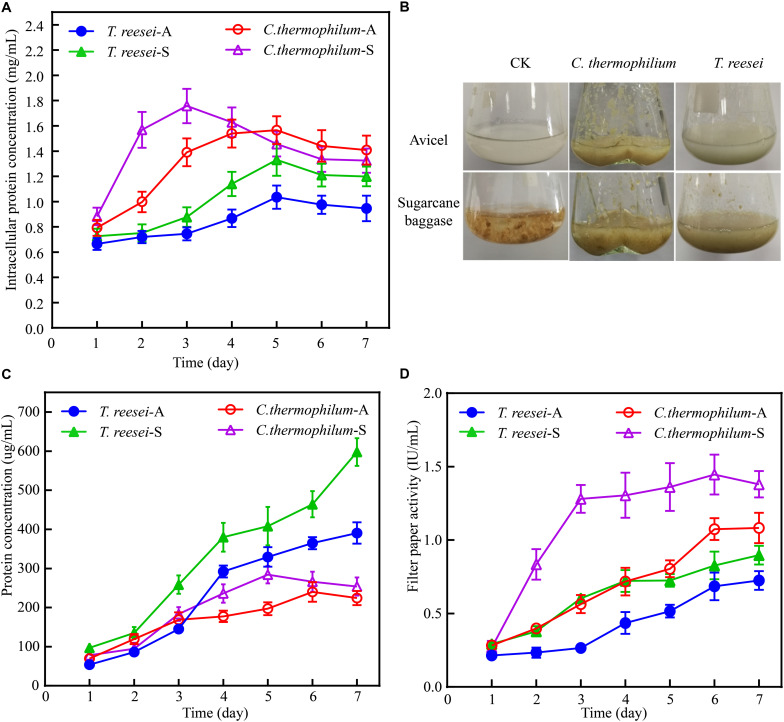
Phenotypic analysis of *C. thermophilum* and *T. reesei* when response to cellulose. **(A)** The intracellular protein concentration of *C. thermophilum* and *T. reesei* mycelia collected from the culture under Avicel or sugarcane bagasse after transferring from the glucose culture. **(B)** Growth phenotype of *C. thermophilum* and *T. reesei* under Avicel or sugarcane bagasse. About 2.2 g wet mycelia of *C. thermophilum* and *T. reesei* were collected and transferred into Avicel or sugarcane bagasse media from glucose contained media and cultivated for 48 h. **(C)** Total secreted protein levels in the supernatants of *C. thermophilum* and *T. reesei* in Avicel or sugarcane bagasse media at different time points. **(D)** Filter paper activities of the supernatants of *C. thermophilum* and *T. reesei* in Avicel or sugarcane bagasse media at different time points. Shown in **(A**,**C,D)** are the mean values of three biological replicates. Error bars show the standard deviations between these replicates. *T. reesei*-A, *T. reesei-S*, *C. thermophilum*-A, and *C. thermophilum*-S respectively indicates *T. reesei* grown on Avicel (A) or sugarcane bassage (S) and *C. thermophilum* grown on Avicel (A) or sugarcane bassage (S). Shown in **(B)** is one of the three represents.

The data shown in Figure 1A demonstrated that the biomass accumulation in *C. thermophilum* was significantly faster than that in *T. reesei* under both Avicel and sugarcane bagasse. Under 48 h of the after-transferring culture, *C. thermophilum* showed obvious growth phenotype under both Avicel and sugarcane bagasse compared to *T. reesei* (Figure 1B), suggesting higher utilization efficiency of cellulose materials in *C. thermophilum* relative to that in *T. reesei*. Further analysis of the extracellular protein levels (Figure 1C) in the culture supernatants of *C. thermophilum* and *T. reesei* showed that under Avicel, the levels of secreted proteins in *C. thermophilum* were slightly higher than that in *T. reesei* in the first 3 days. However, after 3 days, the extracellular protein levels in *T. reesei* were significantly higher than those in *C. thermophilum.* Under sugarcane bagasse, the extracellular protein levels in *T. reesei* were higher than that in *C. thermophilum* in all the tested time points and similar to the observed phenotype under Avicel, the difference of secreted protein levels between *C. thermophilum* and *T. reesei* significantly enlarged after 3 days. These data suggested that the secreted protein levels in the supernatants of *C. thermophilum* were considerably less than those in *T. reesei* when response to both Avicel and sugarcane bagasse. However, analysis of the filter paper activities in the culture supernatants (Figure 1D) showed that consistent with the growth phenotype, *C. thermophilum* exhibited significantly higher filter paper activities compared to *T. reesei* in all the tested time points and under both celluloses carbon sources. This observed phenomenon indicated that *C. thermophilum* might contain cellulases with better specific activities or have better enzyme complex system for cellulose degradation.

### *Chaetomium thermophilum* Contained More Extracellular Protein Categories Compared to *T. reesei*

**FIGURE 2:**
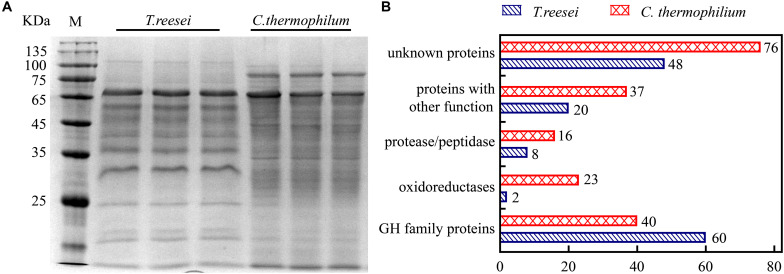
The extracellular protein categories in the supernatants of cellulose cultures were different between *C. thermophilum* and *T. reesei.* **(A)** SDS-PAGE analysis of the secreted proteins in the supernatant of *C. thermophilum* and *T. reesei* in 120-h Avicel cultures after transferring from glucose cultures. **(B)** Major protein categories of the secretome of *C. thermophilum* and *T. reesei* under Avicel. The data was collected by UPLC-MS/MS analysis and the detailed information was listed in Supplementary Tables S2–S7.

To further investigate the mechanism behind the higher cellulose degradation efficiency in *C. thermophilum*, the supernatants of *C. thermophilum* and *T. reesei* cultures under Avicel were analyzed by SDS-PAGE. The data shown in Figure 2A indicated that the bands pattern of the secreted proteins in *C. thermophilum* was quite different from that in *T. reesei.* Although the total extracellular protein levels in *T. reesei* were more than that in *C. thermophilum*, it was worth noting that compared to *T. reesei*, there were more protein bands in the supernatants of *C. thermophilum* cellulose culture, indicating *C. thermophilum* might secrete more protein categories.

Based on the SDS-PAGE analysis, the components and the relative abundances of the extracellular proteins in the supernatants were subsequently evaluated by performing ultra-high performance liquid chromatography coupled tandem mass spectroscopy (UPLC-MS/MS). The data shown in Figure 2B and Supplementary Table S2–S7 further confirmed that the extracellular protein categories were quite different between *C. thermophilum* and *T. reesei.* In *C. thermophilum*, 192 proteins were identified from the UPLC-MS/MS analysis, including 40 glycoside hydrolase proteins, 23 oxidoreductases, 16 protein degradation associated proteins (protease or peptidase) as well as 113 proteins with unknown function or other functions. While in *T. reesei*, 138 proteins were identified including 60 glycoside hydrolase proteins, 8 protease and peptidases, 2 oxidoreductases, and 68 proteins with unknown function or other functions. Unlike *T. reesei*, in which most of the secreted proteins were glycoside hydrolase proteins, *C. thermophilum* contained fewer glycoside hydrolase proteins and much more oxidoreductases, proteases, and peptidases. Considering that most lignocellulases belongs to glycoside hydrolase family proteins, *C. thermophilum* might contain fewer lignocellulases compared to *T. reesei*. Beside containing cellulases with higher specific activities, the different secreted protein categories in *C. thermophilum* might also play an important role in its high-efficiency of degrading celluloses.

### Cellobiohydrolase I From *C. thermophilum* Showed Relatively Higher Specific Activity Compared to *T. reesei* CBH1

**FIGURE 3:**
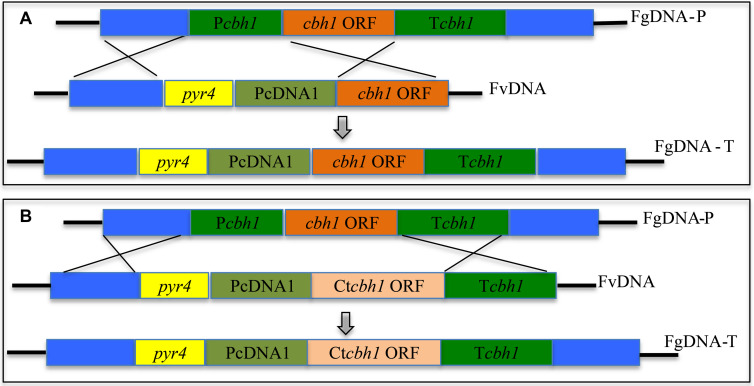
Expression of Tr-CBH1 and Ct-CBH1 in *T. reesei* using cellulase-free background system. **(A)** Schematic diagram of the construction of recombinant strain Tr-cT*cbh1*. **(B)** Schematic diagram of the construction of recombinant strain Tr-cC*cbh1*. FgDNA-P indicates fragments in the parental strain. FvDNA indicates Fragment from plasmid vector constructed for the generation of the indicated recombinant strain. FgDNA-T indicates DNA fragments in the generated transformant strain.

To investigate if *C*. *thermophilum* contain superior cellulases relative to *T. reesei*, one of the major cellulases, CBH1, from *C*. *thermophilum* and *T. reesei* were first characterized and compared. To prevent the effects caused by other cellulases on the measurement of CBH1 activities, a cellulase-free background system (Uzbas et al., 2012) was applied to respectively express *C*. *thermophilum* CBH1 (Ct-CBH1) and *T. reesei* CBH1 (Tr-CBH1) using cDNA1 promoter in *T. reesei* strain Tu6Δ*ku70* under glucose. For the construction of Tr-CBH1 expression strain, the promoter of *cbh1* gene in Tu6Δ*ku70* was replaced with cDNA 1 promoter via homologous recombination (Figure 3A), while for expressing Ct-CBH1, the DNA region including both *cbh1* promoter and the ORF of *cbh1* gene was replaced with cDNA 1 promoter and the cDNA of Ct-CBH1 (Figure 3B). The generated strains were named as Tr-cT*cbh1* and Tr-cC*cbh1*, respectively. Since in the transformants of Tr-cT*cbh1* and Tr-cC*cbh1*, the uridine auxotrophy of Tu6Δ*ku70* strains were complemented by the *pyr4* gene, meanwhile, a uridine complementary strain C*pyr4* was thus constructed by transforming the plasmid pSK-*pyr4* (Qin et al., 2018) into Tu6Δ*ku70* and used as a control for analysis of CBH1 expression.

**FIGURE 4:**
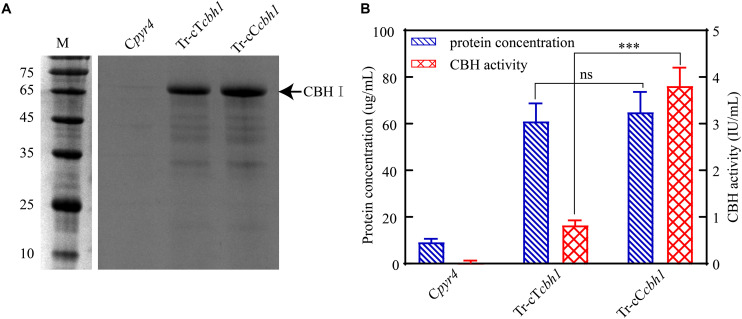
Ct-CBH1 showed higher specific activity compared to Tr-CBH1. **(A)** SDS-PAGE analysis of the secreted proteins in the supernatant of the recombinant *T. reesei* strains C*pyr4*, Tr-cT*cbh1*, and Tr-cC*cbh1*. A suspension of 10^6^ conidia/mL was inoculated into MM media with glucose as the only carbon source and cultivated for 48 h. A total of 20 μL of the supernatant was used for SDS-PAGE analysis. **(B)** Total secreted protein levels and CBH activities in the supernatants of the indicated *T. reesei* strains. The samples used in **(B)** were as same as in **(A)**. Shown are the mean values of three biological replicates. Error bars show the standard deviations between these replicates. The significance of differences between Tr-cT*cbh1* and Tr-cC*cbh1* strains was based on *t*-test analysis by the FDR approach. Asterisks indicate significant differences (^∗∗∗^*P* < 0.001). ns, not significant.

Three independent strains of each Tr-cT*cbh1*, Tr-cC*cbh1*, and C*pyr4* transformants were respectively cultivated in shake flasks with glucose as the only carbon source. The supernatants of 48 h post-inoculation culture were used for analyzing the secreted protein concentration, CBH activities, as well as SDS-PAGE analysis. As shown in Figure 4A, compared to C*pyr4* strains, Tr-cT*cbh1* and Tr-cC*cbh1* strains showed a significantly additional band about 65 kDa. The protein concentration in the supernatants of Tr-cT*cbh1* and Tr-cC*cbh1* cultures was also significantly higher (Figure 4B). Additional enzyme activity analysis (Figure 4B) further confirmed that the recombinant *cbh1* gene from both *C*. *thermophilum* and *T. reesei* were successfully expressed in *T. reesei* under glucose, in which the expression of the native cellulases genes was largely repressed due to the regulation of carbon catabolite repression (CCR). It is worth noting that the expression levels of Ct-CBH1 in Tr-cC*cbh1* strains were slightly higher than Tr-CBH1 in Tr-cT*cbh1* strains (Figure 4A). However, the CBH activity in Tr-cC*cbh1* strains was about 4.7-fold higher than that in Tr-cT*cbh1* strains (Figure 4B). The data that CBH1 activities normalized by total proteins in the supernatant indicated that the relative specific activities of CBH1 from *C*. *thermophilum* were about 4.5-fold higher than CBH1 from *T. reesei*. Although whether other cellulases in *C*. *thermophilum* have higher specific activities still needed to be characterized, the existence of CBH1 with higher specific activity at least confirmed that *C*. *thermophilum* indeed contains superior cellulase.

### *Chaetomium thermophilum* CBH1 Showed Higher Optimum Temperature Compared to *T. reesei* CBH1

**FIGURE 5:**
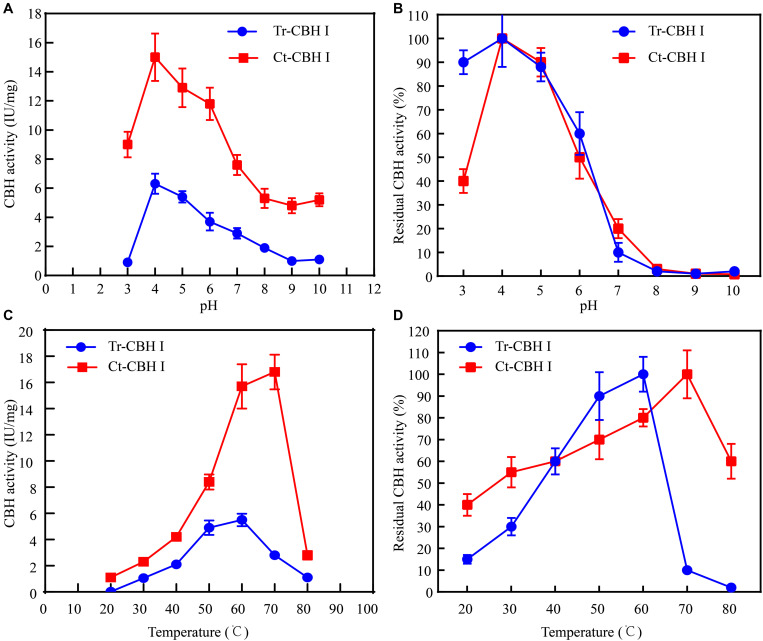
Ct-CBH1 showed higher optimum temperature and better temperature stability compared to Tr-CBH1. **(A)** Optimum pH determination of Tr-CBH1 and Ct-CBH1. **(B)** pH stability analysis of Tr-CBH1 and Ct-CBH1. **(C)** Optimum temperature determination of Tr-CBH1 and Ct-CBH1. **(D)** Temperature stability analysis of Tr-CBH1 and Ct-CBH1. Shown are the mean values of three biological replicates. Error bars show the standard deviations between these replicates.

Considering that *C*. *thermophilum* is a thermophilic fungus, whose optimum growth temperature is around 50°C (Kellner et al., 2016), the optimum temperature of Ct-CBH1 and its temperature stability were subsequently measured and compared with Tr-CBH1. To make sure that the assay for temperature activity curve was carried under the appropriate pH condition, the optimum pH and pH stability for both Ct-CBH1 and Tr-CBH1 were first determined at 50°C. As shown in Figures 5A,B, the optimal pH for both Ct-CBH1 and Tr-CBH1 was pH 4.0, and both Ct-CBH1 and Tr-CBH1 displayed the best residual activities at pH 4.0, suggesting pH 4.0 was the optimum condition for CBH1 activity measurement. The assays for optimum temperature were thus carried at pH 4.0, and at seven different temperatures (20–80°C), by using MUC as the substrate. The result shown in Figure 5C demonstrated that the optimum temperature of Ct-CBH1 was 70°C, while the optimum temperature of Tr-CBH1 was 60°C, and among all the temperatures, Ct-CBH1 displayed higher activities than Tr-CBH1. For the temperature stability analysis (Figure 5D), Tr-CBH1 was stable between 50 and 60°C, the remaining activity dropped dramatically below 50°C or above 60°C. However, Ct-CBH1 was more stable at most of the tested temperatures, which was consistent with reports at previous study (Voutilainen et al., 2008). Taken together, these data indicated that although the enzymatic characteristics of other cellulase genes in C. thermophilum still need to be identified, the above results are at least proved that better cellulases composition system in C. thermophilum was one of the reasons for its higher cellulose-utilizing efficiency relative to T. reesei.

### Replacement of Tr-*cbh1* With Ct-*cbh1* Gene in *T. reesei* Resulted in Increased Production Levels of Most Cellulases Genes

**FIGURE 6:**
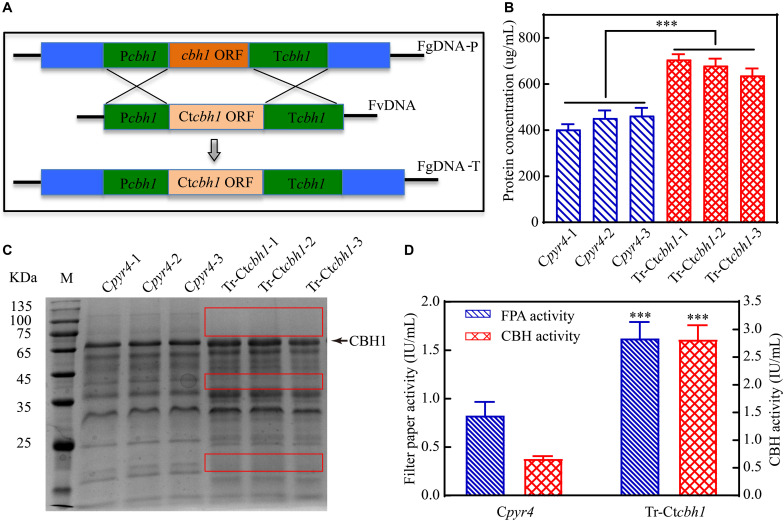
Replacement of Tr-*cbh1* with Ct-*cbh1* gene in *T. reesei* increased the production of cellulases. **(A)** Schematic diagram of the construction of recombinant *T. reesei* strain Tr-Ct*cbh1*. FgDNA-P indicates the fragment in the parental strain. FvDNA indicates the fragment from plasmid vector constructed for generation of the indicated recombinant strain. FgDNA-T indicates DNA fragment in the generated transformant strain. **(B)** Total secreted protein levels of three independent C*pyr4* and Tr-Ct*cbh1* transformants in the supernatant of 120-h Avicel cultures after transferring from glucose cultures. Shown are the mean values of three technical replicates. Error bars show the standard deviations between these replicates. **(C)** SDS-PAGE analysis of the extracellular proteins of C*pyr4* and Tr-Ct*cbh1* strains. The protein bands highlighted with the red frame line indicates proteins in Tr-Ct*cbh1* strains were less than that in C*pyr4* strains. **(D)** Filter paper activities and CBH activities of C*pyr4* and Tr-Ct*cbh1* strains. The samples used in **(C,D)** were as same as in **(B)**. Error bars in **(D)** show the standard deviations between the biological replicates shown in **(C)**. The significance of differences between C*pyr4* and Tr-Ct*cbh1* strains was based on *t*-test analysis by the FDR approach. Asterisks indicate significant differences (^∗∗∗^*P* < 0.001). ns, not significant.

Since the specific activity of Ct-CBH1 was significantly higher than Tr-CBH1, cellobiohydrolase activity in *T. reesei* would be increased if Ct-CBH1 could be expressed in *T. reesei* as a similar level as its native Tr-CBH1. To investigate whether increased cellobiohydrolase activities in *T. reesei* could improve its cellulose utilization, recombinant *T. reesei* strain in which the native *cbh1* gene was replaced with *cbh1* gene from *C*. *thermophilum* was created and named as Tr-Ct*cbh1* (Figure 6A).

Three independent Tr-Ct*cbh1* and C*pyr4* recombinant strains were selected for the measurement of the secreted protein levels, CBH activities, and the filter paper activities under Avicel. It was surprisingly demonstrated that the total secreted protein levels in Tr-Ct*cbh1* strains were about 1.8-fold higher than those in C*pyr4* strains (Figure 6B) and the expression levels of CBH1 in Tr-Ct*cbh1* strains were also significantly higher compared to that in C*pyr4* strains (Figure 6C). The cellobiohydrolase activities in Tr-Ct*cbh1* strains were about 4.1-fold higher than those in C*pyr4* strains due to the higher specific activity of Ct-CBH1. In addition, Tr-Ct*cbh1* strains also displayed around 2.2-fold of increased filter paper activities compared to C*pyr4* strains (Figure 6D).

**FIGURE 7:**
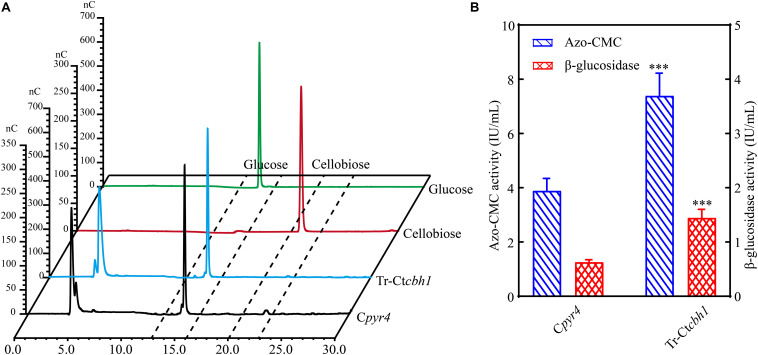
The activities of other cellulases were also increased in Tr-Ct*cbh1* strains. **(A)** HPAEC-PAD analysis of glucose and cellobiose content in the solution of filter paper activity assay (FPA). nC, nano counts, indicating the intensity (abundance) of the signal for each peak. Glucose and cellobiose indicate 100 μM glucose and cellobiose standard sample, respectively. Tr-Ct*cbh1* and C*pyr4* indicate sample taken from the solution of FPA assay with the supernatants of C*pyr4* and Tr-Ct*cbh1* strains under Avicel cultures, respectively. **(B)** Measurements of the endoglucanase activities and β-glucosidase activities of C*pyr4* and Tr-C*tcbh1* strains in the supernatant of 120-h MM+2% Avicel cultures after transferring from 48 h of MM+2% glucose cultures. The significance of differences between C*pyr4* and Tr-Ct*cbh1* strains was based on *t*-test analysis by the FDR approach. Asterisks indicate significant differences (^∗∗∗^*P* < 0.001). ns, not significant.

Increased CBH1 activities in Tr-Ct*cbh1* strains without any change of ß-glucosidase might cause the accumulation of cellobiose when degrading celluloses. Considering that filter paper activity assays were based on the measurement of the abundance of reducing sugars by DNS method (Xiao et al., 2004), thus the increased filter paper activities in Tr-Ct*cbh1* strains might be caused by the accumulation of cellobiose. To prove this, the amount of glucose and cellobiose in the solution of filter paper activity assay (FPA) was determined by high performance anion exchange chromatography with pulsed amperometric detection (HPAEC-PAD). The data shown in Figure 7A unexpectedly revealed that there was no cellobiose accumulation in FPA solutions of both Tr-Ct*cbh1* and C*pyr4* strains, indicating that ß-glucosidase in Tr-Ct*cbh1* strains seemed to be enough to convert the extra cellobiose into glucose. The possible interpretation for this was that more expression of ß-glucosidases might be triggered by the increased CBH1 activity. To investigate whether increased CBH1 activity in *T. reesei* could trigger more expression of other cellulases, besides cellobiohydrolases activities, the activities of ß-glucosidases and endoglucanases of Tr-Ct*cbh1* and C*pyr4* strains under Avicel were measured. As expected, consistent with the above FPA activities data (Figure 6D), the activities of endoglucanase and ß-glucosidase of Tr-Ct*cbh1* strains in 120 h Avicel cultures were 1.9 and 2.3-fold greater than that of C*pyr4* strains (Figure 7B). Taken together, these data suggested that replacement of Tr-CBH1 with Ct-CBH1 in *T. reesei* not only resulted in increasing CBH1 activities, but also lead to more production of other cellulases. Thus we concluded that increased CBH1 activities in *T. reesei* could contribute to improving the cellulose degradation ability in *T. reesei.*

### The Regulation That Increased CBH1 Activities Triggered More Production of Other Cellulases in *T. reesei* Occurred in Transcriptional Level

**FIGURE 8:**
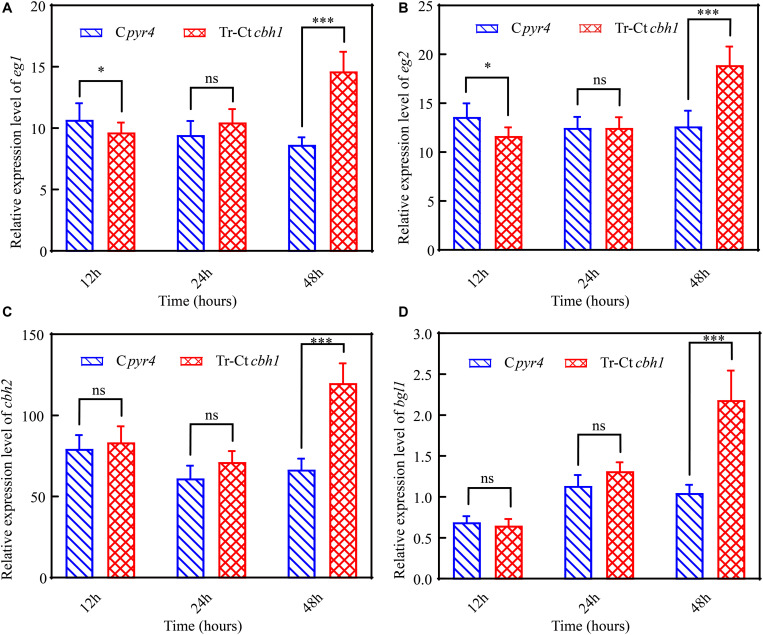
Increased CBH1 activities in *T. reesei* enhanced the induction and expression of the major cellulases genes. Relative quantification of mRNA levels of *eg1* **(A)**, *eg2* **(B)**, *cbh2* **(C)**, and *bgl1* **(D)** genes of C*pyr4* and Tr-Ct*cbh1* strains by qRT-PCR. RNA samples were obtained from the culture of MM+2% Avicel at the indicated time point after a shift of 48 h of MM+2% glucose cultures. The significance of differences between C*pyr4* and Tr-Ct*cbh1* strains was based on *t*-test analysis by the FDR approach. Asterisks indicate significant differences (^∗^*P* < 0.05; ^∗∗∗^*P* < 0.001). ns, not significant.

To further explore the mechanism behind the higher cellulase production in Tr-Ct*cbh1* strains, the transcriptional levels of several major cellulases genes including *eg1* (transcript ID: 122081), *eg2* (transcript ID: 120312), *cbh2* (transcript ID: 72567), and *bgl1* (transcript ID: 76672) under Avicel at different time points were measured by qRT-PCR. As shown in Figure 8, at the time point of 12 h, the expression levels of *eg1* and *eg2* in Tr-Ct*cbh1* strains were slightly lower than that in C*pyr4* strains, and the expression levels of the other two tested genes in Tr-Ct*cbh1* strains were similar as in C*pyr4* strains. However, after induction in cellulose condition for 48 h, all these tested genes in Tr-Ct*cbh1* strains were increased at different extent. The mRNA levels of *eg1* and *eg2* genes in Tr-Ct*cbh1* strains were increased by 1.7 (Figure 8A) and 1.5-fold (Figure 8B) compared to that in C*pyr4* strains, while the mRNA levels of *cbh2* and *bgl1* genes in Tr-Ct*cbh1* strains were 1.8 (Figure 8C) and 2.1-fold (Figure 8D) higher than that in C*pyr4* strains. These data suggested that the more expression of cellulases genes in Tr-Ct*cbh1* strains occurred at transcriptional levels.

**FIGURE 9:**
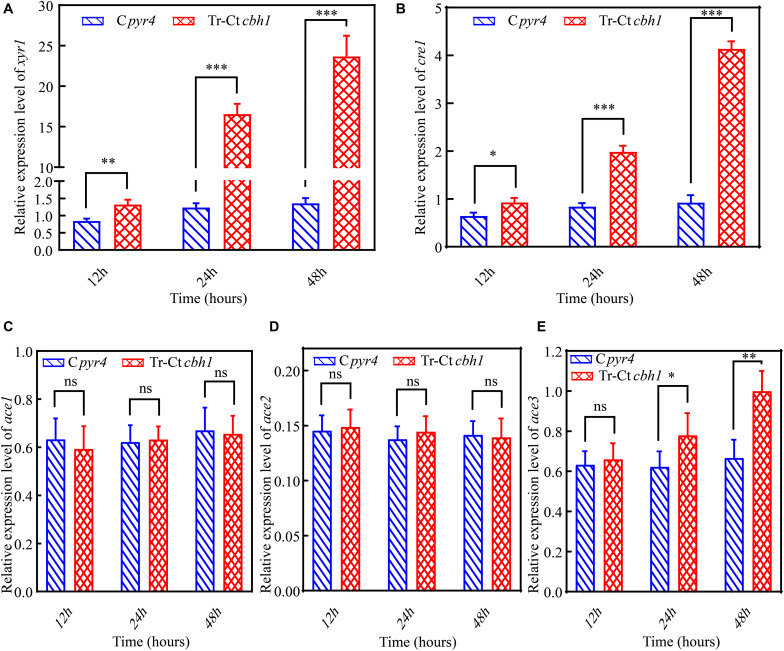
The higher production of cellulases in Tr-Ct*cbh1* strains was caused by increased expression level of *xyr1.* Relative quantification of mRNA levels of *xyr1* **(A)**, *cre1* **(B)**, *ace1* **(C)**, *ace2* **(D)**, and *ace3* **(E)** genes of C*pyr4* and Tr-Ct*cbh1* strains by qRT-PCR. RNA samples were obtained from 12, 24, and 48 h of cultures of MM+2% Avicel after a shift of 48 h of MM+2% glucose cultures. The significance of differences between C*pyr4* and Tr-Ct*cbh1* strains was based on *t*-test analysis by the FDR approach. Asterisks indicate significant differences (^∗^*P* < 0.05; ^∗∗^*P* < 0.01; ^∗∗∗^*P* < 0.001). ns, not significant.

In *T. reesei*, several major TFs including three activators *xyr1*, *ace2* and *ace3*, and two repressors *cre1* and *ace1* were involved in the regulation of the expression of genes encoding lignocellulases genes (Benocci et al., 2017). To investigate if these TFs were involved in the regulation of the increased production of cellulases in Tr-Ct*cbh1* strains, the expression levels of these TFs in Tr-Ct*cbh1* and C*pyr4* strains under Avicel were also measured by qRT-PCR. The data shown in Figure 9 demonstrated that the relative mRNA levels of *xyr1*, *cre1*, and *ace3* in Tr-Ct*cbh1* strains were significantly increased over time compared to that in C*pyr4* strains. At the time point of 48 h, the expression levels of *xyr1* and *cre1* in Tr-Ct*cbh1* strains were about 17.4 and 4.5-fold higher than that in C*pyr4* strains (Figures 9A,B), while the expression levels of *ace3* were only slightly increased by 1.5-fold (Figure 9E). However, the expression levels of *ace1* and *ace2* in Tr-Ct*cbh1* strains exhibited similar levels as that in C*pyr4* strains at all of the time points (Figures 9C,D). Considering that the induction of *xyr1* requires *cre1* (Portnoy et al., 2011), it was reasonable that the expression of *cre1* increased along with *xyr1* here. However, the reason for that the expression level of activator *ace2* was not increased in Tr-Ct*cbh1* strains still needs to be investigated. The data here at least indicated that the major regulator *xyr1* was involved in higher cellulase production in Tr-Ct*cbh1* strains.

## Discussion

Biorefineries include the sustainable processing of plant biomass into a variety of bioproducts and specialty chemicals. Many of these processes require enzymes that are stable at high temperature to enhance mass transfer and reduce substrate viscosity, with a lower risk of contamination. As natural plant-biomass degraders, thermophilic fungi must contain thermostable biomass-degrading enzymes. Therefore, in this study, we evaluated the cellulose-utilizing efficiency of a thermophilic fungus *C*. *thermophilum* and surprisingly found that compared to *T. reesei*, although *C*. *thermophilum* secreted fewer cellulases, it showed higher efficiency to degrade celluloses. This finding indicated that *C*. *thermophilum* might contain cellulases with higher specific activities. The characterization and comparison of Ct-CBH1 and Tr-CBH1 further confirmed this hypothesis. However, UPLC-MS/MS analysis of *C*. *thermophilum* supernatants demonstrated there were many oxidoreductases included in *C*. *thermophilum* protein secretome. Considering that the certain oxidants could make lignocellulose more susceptible for enzymatic degradation (Zhang et al., 2016; Den et al., 2018), the existence of such amounts of oxidoreductases in the supernatants of *C. thermophilum* culture implied that in addition to contain higher efficient cellulases, *C. thermophilum* might display a different mode of action to degrade lignocelluloses.

In *T. reesei*, it has been generally believed that β-glucosidase is a barrier for its cellulose degradation capacity because the extracellular β-glucosidase comprises only about 1% of the total *T. reesei* cellulases complex (Karkehabadi et al., 2014), based on which, many efforts have been conducted to increase enzyme efficiency of hydrolyzing cellulosic substrates by increasing β-glucosidase amount in the cellulases complex from *T. reesei* (Banerjee et al., 2010; Treebupachatsakul et al., 2016). In the opposite, because of the large amount, studies about increasing enzyme efficiency of cellobiohydrolase in *T. reesei* have been rarely reported. In this study, we first reported that increased cellobiohydrolase activities in *T. reesei* could result in a significant increase of its production of other cellulases and cellulose-utilizing efficiency.

It was worth noting that SDS-PAGE analysis of the supernatants of the Tr-Ct*cbh1* and C*pyr4* strains (Figure 6C) showed variations in specific protein levels rather than an overall increase or decrease in the levels of all secreted proteins in Tr-Ct*cbh1* strains compared to that in C*pyr4* strains. Relative to C*pyr4* strains, the expression levels of only a few proteins (highlighted with red frame line in Figure 6C) were decreased, most of the secreted proteins were increased in Tr-Ct*cbh1* strains. Since most of the extracellular proteins in the supernatant of *T. reesei* under cellulose were cellulases and hemi-cellulases (Herpoël-Gimbert et al., 2008), these data suggested that only improved the function of CBH1 in *T. reesei* might lead to increasing the expression levels of most lignocellulose degradation associated genes. The results that the FPA activities (Figure 6D), the Azo-CMC activities and β-glucosidase activities (Figure 7B) in Tr-Ct*cbh1* strains were significantly higher than those in C*pyr4* strains, and besides, the transcriptional levels of *cbh2*, *eg1*, *eg2*, and *bgl1* genes were also higher in Tr-Ct*cbh1* strains (Figure 8), which further proved that the induction and expression of most cellulases genes in *T. reesei* could be improved by only enhancing CBH1 activities.

**FIGURE 10:**
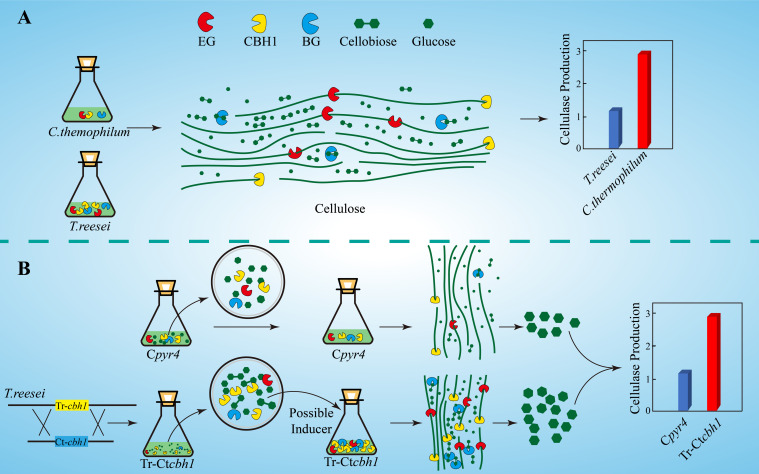
Graphic summary of this study. **(A)** Compared to *T. reesei*, *C. thermophilum* degrades celluloses more efficiently with lower production of cellulases. **(B)** The proposed model of the mechanism for higher production of cellulases in Tr-C*tcbh1* strains. Replacement of Tr-*cbh1* with Ct-*cbh1* gene in *T. reesei* first increased the CBH1 activity, which could cause instantaneous accumulation of cellobiose. This extra cellobiose might function as a possible inducer to trigger more induction and expression of cellulases genes.

The increased expression levels of *xyr1*, *cre1*, and *ace3* in Tr-Ct*cbh1* strains (Figure 9) suggested that an extra inducer might be generated in *T. reesei* when response to cellulose with more cellobiohydrolases in its cellulases system. Cellobiohydrolase is an exocellulase, which cleaves cellulose to release cellobiose from the non-reducing ends. Theoretically, increased cellobiohydrolase in *T. reesei* should result in an accumulation of cellobiose when response to cellulose if the expression level of ß-glucosidase was not correspondingly increased. The higher expression levels of most cellulase genes in Tr-Ct*cbh1* strains could explain why there was no cellobiose accumulation. A possible interpretation for the increased production levels of cellulases in Tr-Ct*cbh1* strains was that the extra cellobiose generated by the increased cellobiohydrolase activities at the very beginning might function as a possible inducer to trigger more expression of cellulases genes (Figure 10). It has been previously reported that cellobiose could induce a low level of cellulase gene expression in *T. reesei* (Vaheri et al., 1979). In addition, sophorose, which is widely viewed as the potent inducer of cellulases in *T. reesei*, also could be generated by the transglycosylation of cellobiose by ß-glucosidase (Sternberg and Mandels, 1979). Furthermore, the growth of *Neurospora crassa* under cellobiose could not induce the expression of celluases genes, however, the deletion of three major β-glucosidase genes in *N. crassa* could result in the expression of cellulases under cellobiose (Znameroski et al., 2012). Moreover, deletion of two intracellular β-glucosidase genes (*cel1a* and *cel1b*) and one extracellular β-glucosidase (*cel3a*) gene in *T. reesei* also could increase the expression levels of cellulases genes when response to cellobiose (Zhou et al., 2012). All of these previous reports suggested that cellobiose played an important role in the induction of cellulases genes, especially when the relative ratio of the cellobiohydrolase and ß-glucosidase was enlarged, which was consistent with the phenomenon observed in this study. Therefore, we speculated that the potential temporary accumulation of cellobiose resulted from the disruption of the synergetic effect of cellobiohydrolase and ß-glucosidase might be able to function as an inducer to trigger the extra induction of cellulases genes in *T. reesei* (Figure10).

## Conclusion

To search for new resources of lignocellulases for plant biomass degradation, here we reported that a thermophilic fungus *C. thermophilum* could degrade celluloses more efficiently with less secreted cellulases compared to *T. reesei*, implying the existence of excellent cellulases genes in its genome. Comparison of the enzyme properties of CBH1 from *C. thermophilum* and *T. reesei* further confirmed this hypothesis. More interesting, our data showed that only raising the function of CBH1 in *T. reesei* could lead to a marked increase in the production levels of other cellulases. Based on the observed phenomenon, we speculated that a certain amount of temporary cellobiose accumulation might play an important role in the process of cellulases genes induction. This study not only provided a new strategy to improve the cellulases yields in *T. reesei*, but also opened the path for fundamental research on thermophilic fungi such as *C. thermophilum*, as well as stimulating investigations into their potential application as sources of commercially important enzymes.

## Data Availability Statement

All datasets generated for this study are included in the article/Supplementary Material.

## Author Contributions

XJ and JD participated in the conception of the study and carried out the majority of the experiments. RH and ZZ were involved in evaluating the cellulose-utilizing efficiency of *C. thermophilum* and *T. reesei*. FQ was involved in mass spectrometry analysis. JH was involved in the project leadership and participated in editing the manuscript. LQ was involved in the conception of the study and participated in the guidance with experimental strategies and technical direction. All authors read and approved the final manuscript.

## Conflict of Interest

The authors declare that the research was conducted in the absence of any commercial or financial relationships that could be construed as a potential conflict of interest.

## Supplementary Material

TABLE S1 Primers used in this study.

TABLE S2 UPLC-MS/MS analysis of protein secretome of *T. reesei* under Avicel (Processed data).

TABLE S3 UPLC-MS/MS analysis of protein secretome of *C. thermophilum* under Avicel (Processed data).

TABLE S4 UPLC-MS/MS analysis of protein secretome of *T. reesei* under Avicel (Raw data of sample 1).

TABLE S5 UPLC-MS/MS analysis of protein secretome of *T. reesei* under Avicel (Raw data of sample 2).

TABLE S6 UPLC-MS/MS analysis of protein secretome of *C. thermophilum* under Avicel (Raw data of sample 1).

TABLE S7 UPLC-MS/MS analysis of protein secretome of *C. thermophilum* under Avicel (Raw data of sample 2).

## Abbreviations

ATCC: American type culture collection
CBH1: cellobiohydrolase I gene
CGMCC: China general microbiological culture collection center
FDR: false discovery rate
FPA: filter paper assay
HPAEC-PAD: ultra-high performance liquid chromatography coupled tandem mass spectroscopy
MM: minimal medium
pNPG: 4-nitrophenyl -D-glucopyranoside
SD: standard deviation
TFs: transcriptional factors
UPLC-MS/MS: ultra-high performance liquid chromatography coupled tandem mass spectroscopy
VMM: Vogel’s minimal medium
YPD: media containing 1% yeast extract 2% peptone 1% dextrose.
