# Supplementary material for: Improved Production of Majority Cellulases in Trichoderma reesei by Integration of cbh1 Gene From Chaetomium thermophilum
Source: Front Microbiol. 2020 Jul 14;11:1633. doi: 10.3389/fmicb.2020.01633 (PMC7381231; doi:10.3389/fmicb.2020.01633)
Supplement: TABLE S1 — Primers used in this study. [file Data_Sheet_1.zip › Table S1.DOCX]

**Table S1. Primers used in this study**

|  | **Primer name** | **Primer sequence (5' to 3')** |
| --- | --- | --- |
|  |  |  |
| **Primers for construction of TrcT*cbh1*** | | |
|  | pTrcTcbh1-F1F | TAGGGAGACCGGCAGCGGCCGCATCTCGCAACACACACAATGG |
|  | pTrcTcbh1-F1R | ATACCCGATATTGCGACTTTGGGGAAAATACACAGAAGACACAAC |
|  | pTrcTcbh1-F2F | TGTTGTGTCTTCTGTGTATTTTCCCCAAAGTCGCAATATCGGGTAT |
|  | pTrcTcbh1-F2R | CATGCGCTGCTACCATCATTGTCTGCAACTGCATCCAAACCATCCT |
|  | pTrcTcbh1-F3F | AGGATGGTTTGGATGCAGTTGCAGACAATGATGGTAGCAGCGCATG |
|  | pTrcTcbh1-F3R | GATGACGGCCAACTTCCGATACATGAGAGAAGTTGTTGGATTGATC |
|  | pTrcTcbh1-F4F | GATCAATCCAACAACTTCTCTCATGTATCGGAAGTTGGCCGTCATC |
|  | pTrcTcbh1-F4R | TTGGAGCACTGCGAGGGGCCAAGAGCGGCGATTCTACGGGTTATG |
|  | pTrcTcbh1-F5F | TAACCCGTAGAATCGCCGCTCTTGGCCCCTCGCAGTGCTCCAATTTTC |
|  | pTrcTcbh1-F5R | CCATTGTGTGTGTTGCGAGATGCGGCCGCTGCCGGTCTCCCTA |
| **Primers for construction of TrcC*cbh1*** | | |
|  | pTrcCcbh1-F1F | GATCAATCCAACAACTTCTCTCATGATGTATAAGAAGTTCGCCGCT |
|  | pTrcCcbh1-F1R | CGTCAGGCTTTCGCCACGGAGCTTTACAGGCACTGGCTGTACCAGG |
|  | pTrcCcbh1-F2F | CCTGGTACAGCCAGTGCCTGTAAAGCTCCGTGGCGAAAGCCTGACG |
|  | pTrcCcbh1-F2R | GAAAATTGGAGCACTGCGAGGGGCCGTTTCGTGCGGCTGAATCCATTC |
|  | pTrcCcbh1-F3F | GAATGGATTCAGCCGCACGAAACGGCCCCTCGCAGTGCTCCAATTTTC |
|  | pTrcCcbh1-F3R | AGCGGCGAACTTCTTATACATCATGAGAGAAGTTGTTGGATTGATC |
|  | **Primers for construction of TrCt*cbh1*** | |
|  | pTrCtcbh1-F1F | CTATAGGGAGACCGGCAGCGGCCGCAAAGCAAGATGTTTGCGATCTAAC |
|  | pTrCtcbh1-F1R | GGCGAACTTCTTATACATCATGATGCGCAGTCCGCGGTTGAC |
|  | pTrCtcbh1-F2F | GTCAACCGCGGACTGCGCATCATGATGTATAAGAAGTTCGCC |
|  | pTrCtcbh1-F2R | CGTCAGGCTTTCGCCACGGAGCTTTACAGGCACTGGCTGTACCAGG |
|  | pTrCtcbh1-F3F | CCTGGTACAGCCAGTGCCTGTAAAGCTCCGTGGCGAAAGCCTGACG |
|  | pTrCtcbh1-F3R | GTTAGATCGCAAACATCTTGCTTTGCGGCCGCTGCCGGTCTCCCTATAG |
|  | **Primers for qRT-PCR** | |
|  | qRTeg1-F | CCCTCAACACTAGCCACCAG |
|  | qRTeg1-R | AGGTCTTGGAGGTGTCAACG |
|  | qRTeg2-F | GCCACTACTATCACCACTTCG |
|  | qRTeg2-R | GTACAGCCAAAGTCAAAACCC |
|  | qRTcbh2-F | ACAAGAATGCATCGTCTCCG |
|  | qRTcbh2-R | TGTTCCACCCGTTGTAGTTG |
|  | qRTbgl1-F | CTGTACATCACCTACCCATC |
|  | qRTbgl1-R | TAGCTGAGATCTCGTCGTC |
|  | qRTxyr1-F | CCATCAACCTTCTAGACGAC |
|  | qRTxyr1-R | AACCCTGCAGGAGATAGAC |
|  | qRTcre1-F | ACACAGCAACCCCAACTCAA |
|  | qRTcre1-R | GAGACGTTGGGAGAGACGAG |
|  | qRTace1-F | GGACGAGGAGGAGATTATG |
|  | qRTace1-R | GTGAGTCTTCTCGTGCTT |
|  | qRTace2-F | GACAAGAAGCTCAGGTGTC |
|  | qRTace2-R | ACTGTGTTCATGGCTGTG |
|  | qRTace3-F | TCGCTGCTTTTGGATCCTGT |
|  | qRTace3-R | GTGCTCAGGAACTCGTCCTC |
